# Directly Acting Antivirals for COVID-19: Where Do We Stand?

**DOI:** 10.3389/fmicb.2020.01857

**Published:** 2020-08-05

**Authors:** Siew L. Teoh, Yi H. Lim, Nai M. Lai, Shaun W. H. Lee

**Affiliations:** ^1^School of Pharmacy, Monash University Malaysia, Subang Jaya, Malaysia; ^2^School of Biosciences, Faculty of Health & Medical Sciences, Taylor’s University, Subang Jaya, Malaysia; ^3^School of Medicine, Faculty of Health & Medical Sciences, Taylor’s University, Subang Jaya, Malaysia; ^4^Asian Centre for Evidence Synthesis in Population, Implementation and Clinical Outcomes (PICO) Health and Well-being Cluster, Global Asia in the 21st Century (GA21) Platform, Monash University Malaysia, Subang Jaya, Malaysia; ^5^School of Pharmacy, Faculty of Health & Medical Sciences, Taylor’s University, Subang Jaya, Malaysia

**Keywords:** rapid review, systematic review, COVID-19, antivirals, pandemic

## Abstract

The outbreak of a novel coronavirus (SARS-CoV-2) in Wuhan, China in December 2019 has now become a pandemic with no approved therapeutic agent. At the moment, the genomic structure, characteristics, and pathogenic mechanisms of SARS-CoV-2 have been reported. Based upon this information, several drugs including the directly acting antivirals have been proposed to treat people with coronavirus disease 2019 (COVID-19). This rapid review aims to describe the directly acting antivirals that have been examined for use in the management of COVID-19. Searches were conducted in three electronic databases, supplemented with a search on arXiv, bioRxiv, medRxiv, ChinaXiv, ClinicalTrials.gov, and Chinese Clinical Trial Registry for studies examining the use of antivirals in COVID-19 to identify for case reports, case series, observational studies, and randomized controlled studies describing the use of antivirals in COVID-19. Data were extracted independently and presented narratively. A total of 98 studies were included, comprising of 38 published studies and 60 registered clinical trials. These drugs include the broad spectrum antivirals such as umifenovir, protease inhibitors such as lopinavir/ritonavir as well as the RNA-dependent RNA polymerase inhibitors, remdesivir, and favipiravir. Other drugs that have been used include the nucleosidase inhibitors and polymerase acidic endonuclease inhibitors which are currently approved for prevention of influenza infections. While some of the drugs appear promising in small case series and reports, more clinical trials currently in progress are required to provide higher quality evidence.

## Introduction

In December 2019, an outbreak caused by a novel coronavirus was reported in Wuhan city, in Hubei province, China. The outbreak was found to be caused by a novel virus, the severe acute respiratory syndrome coronavirus 2 (SARS-CoV-2) (Cheng and Shan, 2020; Wu and McGoogan, 2020). Since then, the cases of SARS-CoV-2 have been reported in every single continent around the world. With over nine million individuals infected with coronavirus disease 2019 (COVID-19) and over 450 thousand death as of mid-June 2020, COVID-19 is now a public health emergency. In many individuals with COVID-19, they often present with a decrease in both CD4^+^ and CD8^+^ T-cells count and suffer from acute respiratory syndrome for 7 to 10 days due to the rapid viral replication (Cheng and Shan, 2020; Zhou et al., 2020a). Clinical features of SARS-CoV-2 infections are similar to SARS-CoV, characterized by fever, dry cough, dyspnoea or shortness of breath, diarrhea, sore throat, muscle ache, and vomiting in some patients (Meo et al., 2020; Wu and McGoogan, 2020).

The SARS-CoV-2 is a member of the family *Coronaviridae*, a positive-sense, single-stranded RNA virus that enters the mammalian cell through an interaction of viral spike glycoprotein that binds to the angiotensin-converting enzyme 2 (ACE_2_) receptor (Fehr and Perlman, 2015). Following receptor binding, the virus uses the host cell receptor and endosome to enter the cell and synthesizes viral polyproteins that encode for the replicase-transcriptase complex. The virus then synthesizes RNA using its RNA-dependent RNA polymerase to synthesize structural proteins leading to completion of assembly and release of viral particles (Fehr and Perlman, 2015; Cheng and Shan, 2020). Genomic sequencing of the virus has revealed that SARS-CoV-2 has a high similarity to the bat-derived SARS-CoV, with approximately 79% identity (Wu C. et al., 2020). Studies have shown that SARS-CoV-2 is spread primarily through the respiratory system and droplets, with an incubation period of between 2 and 14 days, and a median period of 4 days (range, 2–7 days) (Livingston et al., 2020). As such, pharmacological agents that target the spike protein or host’s ACE_2_ proteins used to treat SARS and Middle-East Respiratory Syndrome (MERS) have been suggested as potential agents that could be used to treat patients with COVID-19. Agents proposed to eradicate the coronavirus or at least reduce the effects and hinder the contagion of the SARS-CoV-2 include repurposing currently available drugs such as monoclonal antibodies, antivirals, antimalarial among others (Fehr and Perlman, 2015; Cheng and Shan, 2020).

This intensifying outbreak has led to a surge in registered clinical trials since the infection was first reported (Zhu et al., 2020a). In order to rapidly inform further and better design and conduct of clinical trials, there is an urgent need to provide government agencies on the investigational candidates most suitable for clinical trials. While there are major gaps in knowledge around COVID-19, especially in terms of the effectiveness and safety of various directly acting antiviral agents, a review of the characteristics of published, on-going trials and a synthesis of all available results can help inform current practice and direct future research. This rapid review was performed to provide government bodies on the evidence available in relation to the antiviral drug therapies that have been examined to date.

## Methods

### Search Strategy

We performed a search of PubMed, EMBASE, Cochrane CENTRAL from inception to March 31st, 2020 to search for articles assessing the use of antivirals in patients with SARS-CoV-2 pneumonia without any language restriction. This was supplemented by a search on ClinicalTrials.gov, WHO International Clinical Trials Registry Platform and Chinese Clinical Trial Registry as well as pre-print articles on medRxiv, arXiv, bioRxiv, and ChinaXiv. Keywords used include: novel coronavirus, COVID-19, 2019-nCoV, antivirals, anti-retroviral and humans. Following peer-review, we updated our search to May 31st, 2020 on the database identified previously. We also expanded our keywords to include the following search terms: SARS-Co-v 2, abidol, tenofovir, EIDD-2801, sofosbuvir/ledipasvir, sofosbuvir/daclatasvir.

### Study Selection and Data Abstraction

Articles were screened by two authors (SL, NL, and ST) independently for relevant studies. Studies which described the use of direct acting anti-viral therapies, irrespective of study designs conducted in humans were included. These could include case studies, case reports, cohort studies, observational studies or randomized controlled studies since. *In vitro*, animal studies and reviews were excluded since studies have suggested that these may not directly translate to clinical effects in human. We excluded drugs which does not act directly on virus such as antibiotics and antimalarial since these drugs have limited role in targeting the functions of the virus and preventing it from replicating in the body. All information was extracted independently by authors with discrepancies resolved thorough consensus. Due to the time constraints, the review was not registered in PROSPERO but the corresponding author can be contacted for the full protocol.

### Study Quality and Reporting

The quality of all included studies which were registered and currently underway were assessed subjectively by one author, and classified anecdotally to either low, medium or high. This classification was based upon the study population > study design > sample size of trial and finally the presumed importance of results. Using this approach, a study that reports on patients would be given higher priority over those which had involved healthy subjects. In the event that the study recruited similar populations, a randomized controlled trial would be graded higher priority over a quasi-randomized study > observational study > case series > case report. Finally, a study of similar design that had reported clinical outcomes such as mortality, hospitalization days would be graded higher compared to those which had reported laboratory data only such as presence or absence of SARS-CoV-2 in patients. All data were summarized narratively due to the limited available evidence on the topic.

## Results

**FIGURE 1:**
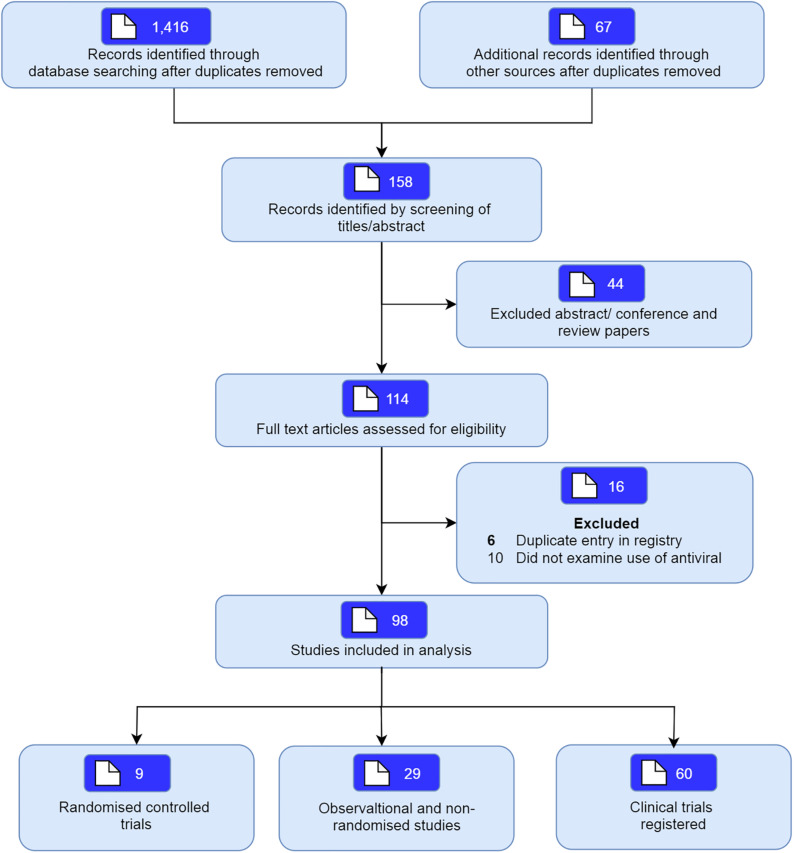
Flow of studies searched and identified.

The database search identified a total of 1,416 articles of which 158 potentially relevant studies were screened. Forty-four studies were excluded based upon screening of abstract, and a further 16 were excluded since they did not include individuals with SARS-CoV-2, or were an *in vitro* studies. A total of 98 studies including nine randomized studies (RCTs) (Beigel et al., 2020; Cao et al., 2020; Chen C. et al., 2020; Goldman et al., 2020; Hung et al., 2020; Li et al., 2020; Lou et al., 2020; Wang Y. et al., 2020; Zheng et al., 2020b) and 29 non-randomized studies (Antinori et al., 2020; Cai et al., 2020; Chen W. et al., 2020; Chen H. et al., 2020; Deng et al., 2020; Gautret et al., 2020; Giacomelli et al., 2020; Grein et al., 2020; Haerter et al., 2020; Holshue et al., 2020; Kim et al., 2020; Lian et al., 2020; Lim et al., 2020; Liu et al., 2020; Panagopoulos et al., 2020; Shi et al., 2020; Vizcarra et al., 2020; Wang Z. et al., 2020; Xi et al., 2020; Xu et al., 2020; Yan et al., 2020; Yang et al., 2020; Ye et al., 2020; Young et al., 2020; Zhang et al., 2020; Zheng et al., 2020a; Zhou et al., 2020b; Zhu et al., 2020b; Zuo et al., 2020) examining the use of antivirals in COVID-19 were included (Figure 1). We also included another 60 registered clinical trials which were at clinical phases 2, 3, or 4 (Supplementary Appendix Table 1, Supplementary Appendix Figure 1, and Supplementary Appendix Figure 2). Most of the trials will be mainly conducted in China but also from other countries including France, Canada, Hong Kong, Iran, Brazil, Egypt, Pakistan, Thailand, United States, Spain, and Korea. The pharmaceutical interventions found for COVID-19 treatment include remdesivir, oseltamivir, favipiravir, danoprevir, ritonavir, darunavir, baloxavir marboxil, azvudine, triazavirin, umifenovir, lopinavir either alone or in combination with other products such as human immunoglobulin, interferons, carrimycin, bevacizumab, cobicistat, and traditional Chinese medicines (see Tables 1, 2 for characteristics of studies identified).

**TABLE 1:** Study characteristics and reported outcomes from randomized controlled studies.

Study ID, Country	Study design	Disease severity	Primary outcome	Efficacy outcomes	Safety outcomes, *n* (%)
Cao et al. (2020) China	Open label single center RCT, *n* = 199 Int: Lopinavir–ritonavir (400 mg and 100 mg) twice daily for 14 days with standard care Ctr: Standard care comprising of as necessary supplemental oxygen, non-invasive and invasive ventilation, antibiotic agents, vasopressor support, renal-replacement therapy, and extracorporeal membrane oxygenation (ECMO)	Severe	Time to clinical improvement on two points (from randomization) on the following seven scale category or live discharge 1. Not hospitalized with resumption of normal activities; 2. Not hospitalized, but unable to resume normal activities 3. Hospitalized, not requiring supplemental oxygen 4. Hospitalized, requiring supplemental oxygen 5. Hospitalized, requiring nasal high-flow oxygen therapy, non-invasive mechanical ventilation, or both 6. Hospitalized, requiring ECMO, invasive mechanical ventilation, or both 7. Death	Median time to clinical improvement Int: 15 days Ctr: 16 days Hazard ratio: 1.39, 95% CI: 1.00–1.91 Mortality Int: 19 (19.2%) Ctr: 25 (25.0%) MD: −5.8%; 95% CI: −17.3 to 5.7. Median ICU length of stay Int: 6 (2 to 11) Ctr: 11 (7 to 17) MD: −5 days; 95% CI: −9 to 0 Number with clinical improvement at 14 days Int: 45 (45.5) Ctr: 30 (30.0) MD: −15.5; 95% CI: 2.2 to 28.8 Median hospital stay Int: 14 (12 to 17) Ctr: 16 (13 to 18) MD: 1, 95% CI: 0 to 2	Any adverse event (any grade), *n* (%) Int: 46 (48.4) Ctr: 49 (49.5) Any adverse event (Grade 3 or 4), *n* (%) Int: 20 (21.1) Ctr: 11 (11.1) Serious adverse event (any grade), *n* (%) Int: 19 (20.0) Ctr: 32 (32.3) Serious adverse event (Grade 3 or 4), *n* (%) Int: 17 (17.9) Ctr: 31 (31.3)
Chen C. et al. (2020) China ChiCTR2000030254	Open label multi-center RCT, *n* = 240 Int: Favipiravir 1600 mg twice daily on day 1, then 600 mg twice daily for 7–10 days with standard care Ctr: Umifenovir 200 mg three times daily for 7–10 days with standard care	Mild and moderate	Clinical recovery defined as • Normal body temperature for more than 3 days, with axillary temperature ≤ 36.6°C • Respiratory rate ≤ 24 times/min • Oxygen saturation ≥ 98% • Mild or no cough	Clinical recovery at day 7, *n* (%) Favipiravir: 71 (61.2%) Umedipavir: 62 (51.7%)	Adverse effect (all) Total patients, *n* (%) Favipiravir : 37 (31.9) Umedipavir: 28 (23.3) Total events, *n* Favipiravir : 43 Umedipavir: 33
Li et al. (2020) China NCT04252885	Open label single-centre RCT, *n* = 86 Int 1: Lopinavir–ritonavir (400 mg and 100 mg) twice daily for 7–14 days with standard care and oxygen therapy if needed Int 2: Umifenovir 200 mg three times daily for 7–14 days with standard care and oxygen therapy if needed Ctr: Standard care and oxygen therapy if needed (no antivirals)	Mild and moderate	Time to negative detection of SARS-CoV-2 nucleic acid at day 21	Mean time to negative conversion of SARS-CoV-2, mean (SD) Lopinavir/ritonavir: 9.0 (5.0) Umifenovir: 9.1 (4.4) Ctr: 9.3 (5.2) Difference between group: *p* = 0.98 Rate of positive to negative conversion at day 7, total patients, *n* (%) Lopinavir/ritonavir: 12 (35.3) Umifenovir: 13 (37.1) Ctr: 7 (41.2) Rate of positive to negative conversion at day 14, total patients, *n* (%) Lopinavir/ritonavir: 29 (85.3) Umifenovir: 32 (91.4) Ctr: 13 (76.5)	Adverse effect (all) Total patients Lopinavir/ritonavir: 12 (35.3) Umifenovir: 5 (14.3) Ctr: 0 (0) Serious adverse effect (all) Total patients Lopinavir/ritonavir: 1 (2.9) Umifenovir: 0 (0) Ctr: 0 (0)
Wang Z. et al. (2020) China NCT04257656	Multi-center RCT, *n* = 237 Int: Remdesivir 200 mg loading dose on day 1 is given, followed by 100 mg iv once-daily maintenance doses for 9 days ± concomitant use of lopinavir–ritonavir, interferons, or corticosteroids Ctr: Placebo for 10 days ± concomitant use of lopinavir–ritonavir, interferons, or corticosteroids	Severe	Time to clinical improvement within 28 days after randomization	Time to clinical improvement, median (IQR) Remdesivir: 21 (13–28) Placebo: 23 (15–28) Early symptom resolution, *n* (%) Remdesivir: 8 (11) Placebo: 7 (15) Clinical improvement rates Day 7, *n* (%) Remdesivir: 4 (3) Placebo: 2 (2) Day 14 Remdesivir: 42 (27) Placebo: 18 (23) Day 28 Remdesivir: 103 (65) Placebo: 45 (58) Duration of mechanical ventilation in days, median (IQR) Remdesivir: 7.0 (4.0–16.0) Placebo: 15.5 (6–21.0) 28 days mortality, *n* (%) Remdesivir: 22 (14) Placebo: 10 (13)	Adverse events, *n* (%) Remdesivir: 102 (66) Placebo: 50 (64) Severe adverse events, *n* (%) Remdesivir: 28 (18) Placebo: 9 (6)
Beigel et al. (2020) NCT04280705	Multi-center RCT in Europe, Asia, and America, *n* = 1,107 Int: Remdesivir 200 mg loading dose on day 1 is given, followed by 100 mg iv once-daily maintenance doses for 9 days Ctr: Placebo for 10 days	Moderate to severe	Time to recovery, defined as the first day, during the 28 days after enrolment, on which a patient satisfied categories 1, 2, or 3 on the eight-category ordinal scale.	Time to recovery, median (95% CI) Remdesivir: 11 (9–12) Placebo: 15 (13–19) No of recoveries, *n* (%) Remdesivir: 334 (63.3) Placebo: 273 (52.4) Mortality at day 14, *n* (%) Remdesivir: 32 (5.9) Placebo: 54 (10.4)	Adverse events, *n* (%) Remdesivir: 156 (28.8) Placebo: 172 (33.0) Severe adverse events, *n* (%) Remdesivir: 114 (21.1) Placebo: 141 (27.0)
Goldman et al. (2020) NCT04292899	Open label multi-center RCT in Europe, Asia and America, *n* = 397 Int: Remdesivir 200 mg loading dose on day 1 is given, followed by 100 mg iv once-daily maintenance doses for 4 days Ctr: Remdesivir 200 mg loading dose on day 1 is given, followed by 100 mg iv once-daily maintenance doses for 4 days	Moderate to severe	Clinical status on day 14, assessed on a 7-point ordinal scale on the following 1, death; 2, hospitalized, receiving invasive mechanical ventilation or ECMO; 3, hospitalized, receiving non-invasive ventilation or high-flow oxygen devices; 4, hospitalized, requiring low-flow supplemental oxygen; 5, hospitalized, not requiring supplemental oxygen but receiving ongoing medical care (related or not related to COVID-19); 6, hospitalized, requiring neither supplemental oxygen nor ongoing medical care (other than that specified in the protocol for remdesivir administration); and 7, not hospitalized	Time to clinical improvement, median Remdesivir 5 days: 10 Remdesivir 10 days: 11	Adverse events, *n* (%) Remdesivir 5 days: 141 (70) Remdesivir 10 days: 145 (74) Severe adverse events, *n* (%) Remdesivir 5 days: 42 (21) Remdesivir 10 days: 68 (35)
Hung et al. (2020) Hong Kong NCT04276688	Multicenter, open label RCT, *n* = 127 Int: Lopinavir–ritonavir (400 mg and 100 mg) twice daily, ribavirin 400 mg twice daily and three doses of eight million iu interferon beta-1b on alternate days for three doses Ctr: Lopinavir–ritonavir (400 mg and 100 mg) twice daily for 7–14 days	Unclear	Time to achieve a negative RT-PCR result for SARS-CoV-2 in a nasopharyngeal swab sample.	Time to achieve negative RT-PCR result for SARS-CoV-2, median days (IQR) Int: 7 (5–11) Ctr: 12 (8–15) Hospital stay, median days (IQR) Int: 9.0 (7.0–13.0) Ctr: 14.5 (9.3–16.0)	Adverse events, *n* (%) Int: 41 (48) Ctr: 20 (41) Severe adverse events, *n* (%) Int: 0 (0) Ctr: 1 (2)
Zheng et al. (2020) China ChiCTR2000029496	Open label single-center RCT, *n* = 89 Int 1: Novaferon (20 μg) twice daily Int 2: Novaferon (20 μg) twice daily + lopinavir/ritonavir (400 mg and 100 mg) twice daily Ctr: Lopinavir–ritonavir (400 mg and 100 mg) twice daily	Moderate to severe	SARS-CoV-2 clearance rates in COVID-19 patients assessed on day 6 of antiviral treatment.	SARS-CoV-2 clearance at day 3, *n* (%) Int 1: 5 (16.3) Int 2: 11 (36.7) Ctr: 3 (10.3) SARS-CoV-2 clearance at day 6, *n* (%) Int 1: 15 (50.0) Int 2: 18 (60.0) Ctr: 7 (24.1) SARS-CoV-2 clearance at day 9, *n* (%) Int 1: 17 (56.7) Int 2: 21 (70.0) Ctr: 15 (51.7) Median time to SARS-CoV-2 clearance, days Int 1: 6 Int 2: 6 Ctr: 9	Adverse events, *n* (%) Int 1: 0 (0) Int 2: 3 (10.0) Ctr: 4 (13.8) Severe adverse events, *n* (%) Int 1: 0 (0) Int 2: 0 (0) Ctr: 0 (0)
Lou et al. (2020) China ChiCTR2000029544	Open label single-center RCT, *n* = 89 Int 1: Antiviral therapy + baloxavir marboxil 80 mg daily for 4 days and on day 7 if needed Int 2: Antiviral therapy + favipiravir with loading dose of 1600 mg followed by 600 mg three times daily up to 14 days Ctr: current antiviral treatment (drug, dose and frequency not stated)	Unclear	Number of people with viral negative at day 14 Time to clinical improvement defined as 2 point improvement on a seven-category ordinal scale or live discharge from the hospital	Viral negative at day 14, *n* (%) Int 1: 7 (70) Int 2: 7 (77) Ctr: 10 (100) Time to clinical improvement median days, (IQR) Int 1: 14 (6–49) Int 2: 14 (6–38) Ctr: 14 (6–49)	Adverse events, *n* (%) Int 1: 10 (100) Int 2: 8 (88) Ctr: 9 (90) Severe adverse events, *n* (%) Int 1: 0 (0) Int 2: 0 (0) Ctr: 0 (0)

*Ctr, control; Int, intervention; MD, difference; 95% CI, 95% confidence interval.*

**TABLE 2:** Summary of reported clinical effects on use of antivirals from non-randomized studies.

Study ID, Country	Study design	Disease severity	Efficacy outcomes	Safety outcomes, n (%)
**Prospective Open-label/Cohort Study**				
Antinori et al. (2020) Italy	Prospective open-label study, *n* = 35 Int: Remdesivir (compared between patients in intensive care unit and infectious diseases ward)	Severe	Intensive care unit patients: By 10 days of treatment, 4/18 (22.2%) of patients improved in hospitalization Status (1 not requiring supplemental oxygen and 3 weaned from invasive ventilation), 10/18 (55.5%) still undergoing invasive ventilation, and 4/18 (22.2%) died; By the 28 days of follow-up, 7/18 (38.9%) of patients improved in hospitalization Status (6 discharged, 1 weaned from invasive ventilation), 16.7% still undergoing mechanical ventilation and the other 44.4% died. Infectious diseases ward patients: By 10 days of treatment, 6/17 (35.3%) of patients improved in hospitalization Status (1 discharged, 3 no longer required oxygen supplementation, 2 no longer required high-flow therapy and/or non-invasive mechanical ventilation); 10 still required high-flow therapy and/or non-invasive mechanical ventilation, and 1 died. By day 28 of follow-up, hospitalization status had improved in 88.2% of the IDW patients (14 had been discharged, one no longer required oxygen supplementation) but one still required high-flow therapy and/or non-invasive mechanical ventilation.	Severe adverse advents: Hypertransaminasemia 15/35 (42.8%) Increased total bilirubin levels 7/35 (20.0%) Acute kidney injury 8/35 (22.8%) Rash 2/35 (5.7%) Any adverse event leading to treatment discontinuation 8/35 (22.8%)
Cai et al. (2020) China ChiCTR2000029600	Prospective open-label, non-randomized, *n* = 80 Int: Favipiravir 1600 mg twice daily on day 1, 600 mg twice daily from day 2–14 Ctr: Lopinavir–ritonavir (400 mg and 100 mg) twice daily for up to 14 days		Median days to viral clearance, (IQR) Int: 4 (2.5–9) Ctr: 11 (8–13) *P* < 0.001 Improvement in chest CT scans at day 14, *n* (%) Int: 32 (91.4) Ctr: 28 (62.2) *P* = 0.004	Adverse effect (all), *n* (%) Int: 4 (11.4) Ctr: 25 (55.6)
Chen W. et al. (2020) China	Prospective open-label study, *n* = 62 Int: Arbidol Ctr: Standard of care including interferon antiviral treatment	NR	Hospitalization period in the test group and control group: (16.5 ± 7.14) days and (18.55 ± 7.52) days Fever and cough in the test group were relieved markedly faster than those in the control group (*p* < 0.05); time for two consecutive negative nucleic acid tests in the test group were shorter than that in the control group.	No significant difference between the two groups for any adverse drug reaction.
Grein et al. (2020) United States, Japan, Europe, Canada	Prospective cohort study, *n* = 53 Int: Remdesivir 200 mg on day 1, then 100 mg daily for the following 9 days.	NR	Over a median follow up of 18 days (IQR 13–23) after receiving the first dose of remdesivir, 36/53 (68%) showed improvement in oxygen support, 8/53 (15%) showed worsening. By the date of most recent follow up, 25/53 (47%) had been discharged. By 28 days of follow-up, cumulative incidence of clinical improvement was 84% (95% CI 70–99). Clinical improvement was less frequent among those receiving invasive ventilation than among those receiving non-invasive oxygen support (HR 0.33; 95% CI 0.16–0.68) and among patients 70 years and older as compared to patients younger than 50 years (HR 0.29; 95% CI 0.11–0.74). 7/53 patients (13%) died after the completion of remdesivir treatment. Overall mortality from the date of admission was 0.56 per 100 hospitalization days (95% CI 0.14–0.97) and did not differ among patient receiving invasive ventilation and non-invasive oxygen support. Hazard ratio for patient receiving invasive ventilation as compared with patient receiving non-invasive oxygen support was 2.78 (95% CI 0.33–23.19). Mortality rate was higher among patients 70 years and older as compared with patients younger than 70 years (HR 11.34; 95% CI 1.36–94.17) and among those with higher serum creatinine at baseline (HR 191; 95% CI 1.22–2.99).	32 patients (60%) reported adverse events during follow up. Most common adverse events were increased hepatic enzymes, diarrhea, rash, renal impairment, and hypotension. 12 patients (23%) had serious adverse events, which all received invasive ventilation at baseline. Most common serioys adverse events were multiple organ dysfunction syndrome, septic shock, acute kidney injury, and hypotension. 4 patients (8%) discontinued remdesivir prematurely, due to worsening of pre-existing renal failure (*n* = 1), multiple organ failure (*n* = 1), elevated aminotransferases (*n* = 1), including one patient with a maculopapular rash.
**Retrospective cohort studies**				
Deng et al. (2020) China ChiCTR2000030254	Retrospective cohort, *n* = 33 Int: Umifenovir 200 mg three times daily and lopinavir–ritonavir (400 mg and 100 mg) twice daily for 5–12 days Ctr: Lopinavir–ritonavir (400 mg and 100 mg) twice daily for 5–12 days	Moderate to severe	Negative SARS-CoV-2 detection at day 7, *n* (%) Int: 12 (75) Ctr: 6 (35) *p* < 0.05 Negative SARS-CoV-2 detection at day 14, *n* (%) Int: 15 (94) Ctr: 9 (53) *p* < 0.05 Improvement in chest CT scans at day-7 Int: 11 (69) Ctr: 5 (29) *p* < 0.05	Adverse effect (all) Total patients, *n* (%) Favipiravir : 37 (31.9) Umedipavir: 28 (23.3) Total events, *n* Favipiravir : 43 Umedipavir: 33
Giacomelli et al. (2020) Italy	Retrospective intent-to-treat analysis, *n* = 172 Lopinavir/ritonavir (LPV/r) + hydroxychloroquine (HCQ): Int: Treatment started within 5 days of symptom onset (early treatment) (25% of patients) Ctr: Treatment started later (delayed treatment) (75% of patients)		Rate of clinical improvement increased over time to 73.3% on day 30, without any significant difference between the two groups (Gray’s test *P* = 0.213). No significant association between the timing of the start of treatment and the probability of 30-day mortality (adjusted odds ratio [aOR] early treatment *vs* delayed treatment = 1.45, 95% confidence interval 0.50–4.19).	8% of the patients discontinued the treatment because of severe gastrointestinal disorders attributable to LPV/r.
Kim et al. (2020) Korea	Retrospective cohort study, *n* = 65 Lopinavir–ritonavir 400 mg/100 mg twice daily (*n* = 31) Hydroxychloroquine 400 mg once daily (*n* = 34)		Median duration of treatment was 7 days Median time to negative conversion of viral RNA Lopinavir–ritonavir: 21 days Hydroxychloroquine: 28 days Lopinavir–ritonavir (aHR 2.28; 95% CI 1.24–4.21) and younger age (aHR 2.64; 95%CI 1.43–4.87) were associated with negative conversion of viral RNA. No significant difference in time to clinical improvement between lopinavir–ritonavir-treated patients and hydroxychloroquine-treated patients (median 18 days vs. 21 days).	Lymphopenia and hyperbilirubinemia were more frequent in lopinavir–ritonavir group compared with hydroxychloroquine group. One serious adverse event (ARDS) occurred in one patient treated with lopinavir–ritonavir, two serious adverse events (ARDS and shock) occurred in patients treated with hydroxychloroquine.
Lian et al. (2020) China	Retrospective cohort, *n* = 81 Int: Umifenovir 0.2 g three times a day + symptomatic treatment Ctr: symptomatic treatment	Moderate and severe	Rate of negative pharyngeal swab tests for SARS-CoV-2 within 1 week after admission: Int: 33 (73%) Ctr: 28 (78%) Time from admission to first negative test of SARS-CoV-2: Int: 6 days (4–8) Ctr: 3 days (1–7) Time from onset of symptoms to first negative test of SARS-CoV-2: Int: 18 days (12–21) Ctr: 16 days (11–21) Length of hospital stay: Int: 13 days (9–17) Ctr: 11 days (9–14)	5/45 (45%) patients in Umifenovir group and 3/36 (8%) in control group demonstrated digestive symptoms, including diarrhea and nausea.
Panagopoulos et al. (2020) Greece	Retrospective cohort, *n* = 16 Group A: Lopinavir/ritonavir + hydroxychloroquine + azithromycin Group B: Hydroxychloroquine + azithromycin	NR	7/8 patients in group A recovered, one needed intubation and mechanical ventilation. 1/8 patient in group B recovered, 3/8 died, 4/8 patients needed intubation. Days of hospitalization: Group A: 14.71 ± 0.76 Group B: 11.40 ± 2.07 Days for clinical improvement (no fever): Group A: 6.00 ± 1.16 Group B: 4.4 ± 1.52 Days for negative result of RT-PCR for SARS-CoV-2: Group A: 8.86 ± 1.68 Group B: 13.8 ± 2.68	NR
Shi et al. (2020) China	Retrospective cohort study, total *n* = 184 (divided into seven groups). Symptomatic treatment group, Arbidol group, lopinavir/ritonavir group, Arbidol + lopinavir/ritonavir group, interferon group, interferon + lopinavir/ritonavir group, and interferon + darunavir group (Doses: interferon, interferon-α2β (aerosol inhalation), 100,000 U/kg, 2 times/day; Arbidol, 200 mg, 3 times/day; lopinavir/ritonavir, 2 tablets, 2 times/day; darunavir, 1 tablet, 1 time/day)	Not classified	Data extensive among seven groups, but no significant different among groups in the rates of pneumonia resolution and length of hospital stay. Pneumonia resolution after treatment, *n* (%) Int 1: 16 (53%) Int 2: 12 (44%) Int 3: 9 (36%) Int 4: 24 (59%) Int 5: 16 (76%) Int 6: 14 (61%) Ctr: 7 (41%) Length of hospital stay, mean ± SD Int 1: 15.7 days ± 6.4 Int 2: 18.4 ± 7.2 Int 3: 18.5 ± 9.5 Int 4: 16.5 ± 5.5 Int 5: 16.2 ± 7.1 Int 6: 17.4 ± 7.0 Ctr: 20.0 ± 6.0	NR
Vizcarra et al. (2020) Spain	Prospective cohort, *n* = 51 HIV-infected individuals diagnosed with COVID-19. Nine individual received protease inhibitor before COVID-19, 37 individuals received tenofovir before COVID-19 *N* = 39 HIV-infected individuals received off-label treatment for COVID-19. - Hydroxychloroquine (*n* = 30) - Azithromycin (*n* = 19) - Ritonavir/lopinavir (*n* = 14) - Tocilizumab (*n* = 4) - Systemic corticosteroids (*n* = 15)	Mild, moderate and severe	Clinical outcomes for HIV-infected COVID-19 individuals (*n* = 51): Respiratory failure, *n* (%) Mild or moderate: 4 (11%) Severe: 13 (100%) Sepsis, *n* (%) Mild or moderate: 2 (5%) Severe: 9 (69%) Critical disease or intensive care unit admission, *n* (%) Mild or moderate: 0 Severe: 6 (46%) Invasive mechanical ventilation, *n* (%) Mild or moderate: 0 Severe: 5 (38%) Death, *n* (%) Mild or moderate: 0 Severe: 2 (15%) Recovered, *n* (%) Mild or moderate: 35 (92%) Severe: 9 (69%) Duration of hospital stay, days Mild or moderate: 8 (6–17) Severe: 8 (6–19)	NR
Xu et al. (2020) China	Retrospective cohort, multi-center study (*n* = 141). Combined group (*n* = 71) patients were given Arbidol and IFNa2b Monotherapy group (*n* = 70): patients inhaled IFNa2b for 10 to 14 days.	Mild and moderate (non-ventilated)	The median hospitalization days was 27.1 vs. 24.2 days in two group (*P* = 0.056). After treatment for 7 to 14 days, there was no statistically differences of the viral RNA clearance days between two groups.	There were no differences between the two groups in hemoglobin, WBC count, platelet count, ALT, AST, or creatinine during or after treatment. Thirteen patients (18.8%) treated with Arbidol demonstrated mild nausea, stomachache, but all patients could tolerate without giving up treatment.
Yan et al. (2020) China	Retrospective cohort, *n* = 120 Int: Lopinavir–ritonavir (400 mg and 100 mg) twice daily for 10 or more days Ctr: Standard care	Mild, moderate, severe, and critical	Median duration of treatment was 10 days (IQR: 9–10 days Median duration of SARS-CoV-2 shedding, (IQR) Int: 22 (18–29) Ctr: 28.5 (19.5–38) *p* = 0.02	NR
Yang et al. (2020) China	Retrospective cohort, single-center study involving frontline health professionals (*n* = 164), including 82 infected with COVID-19 and 82 uninfected controls. Arbidol were taken by 23.2% if the participants in the infected group and 58.5% of the participants in the uninfected group as prophylaxis against symptomatic COVID-19 requiring hospital admission.	Asymptomatic infected and uninfected groups.	The cumulative uninfected rate of health professionals in the Arbidol group was significantly higher than that of individuals in the non-Arbidol group (log-rank test, χ2 = 98.74; *P* < 0.001). Forty-eight patients (58.5%) in the infection group were hospitalized, with a median age of 39 (31–49) years, of whom 7 (14.6%) were prophylactically administered Arbidol.	NR
Ye et al. (2020) China	Retrospective cohort, *n* = 47. Lopinavir/Ritonavir along with Arbidol and interferon (*n* = 42), and “control” (no lopinavir/ritonavir, with Arbidol and interferon only, *n* = 5). “The per ml of LPV/r oral liquid contained 80 mg lopinavir and 20 mg ritonavir. Usage and dosage: 5 ml/time (400/100 mg) for adults, twice a day or 10 ml/time (800/200 mg) once a day with food”	Not classified	“Compared with the control group, the patients in the test group returned to normal body temperature in a shorter time (test group: 4.8 ± 1.94 days vs. control group: 7.3 ± 1.53 days, *p* = 0.0364).” No significant differences between groups otherwise.	The abnormal percentage of ALT and AST in the test group was lower than that in the control group.
Zheng et al. (2020) China	Retrospective cohort, *n* = 55 Mild: Intermitted low-flow oxygen therapy (≤3 L/min) and antiviral treatment for 10 days Moderate: continuous middle-flow oxygen therapy (3∼5 L/min), triple antiviral treatment, ribavirin 500 mg twice daily and recombinant interferon-α2b (5 million units) twice daily for 10 days Severe: Oxygen support including mask oxygen (>5 L/min), high flow nasal oxygen therapy (HFNO), or non-invasive ventilation (NIV), triple antiviral treatment, ribavirin and recombinant interferon-α2b (5 million units) twice daily for 10 days. All patients also received methylprednisolone (0.5∼1 mg/kg/d × 5 days). Empirical antibiotic treatment given if bacteria infection was suspected. Treatment-failure patients were prepared early for intubation and invasive mechanical ventilation and considered for ECMO	Mild, moderate and severe	Improvement in clinical symptoms, *n* (%) Non-severe (mild/moderate cases): 31 (91.2) Severe: 18 (85.7) *p* = 0.85 At least 50% improvement in chest CT scans at 7 days, *n* (%) Non-severe: 22 (64.7) Severe: 12 (57.4) At least 75% improvement in chest CT scans at 14 days, *n* (%) Non-severe: 28 (82.4) Severe: 16 (76.2) Negative SARS-CoV-2 detection, *n* (%) Non-severe: 33 (97.1) Severe: 20 (95.2) *P* = 0.92	NR
Zhu et al. (2020) China	Retrospective cohort, *n* = 50 Lopinavir/ritonavir group received 400 mg/100 mg twice a day for a week Umedipavir 0.2 g Arbidol three times a day	NR	Negative SARS-CoV-2 detection at day 7, *n* (%) Lopinavir/ritonavir: 8 (23.5) Umedipavir: 8 (50) Negative SARS-CoV-2 detection at day 14, *n* (%) Lopinavir/ritonavir: 19 (55.9) Umedipavir: 16 (100)	Adverse event, all, *n* (%) Lopinavir/ritonavir: 4 (11.8) Umedipavir: 6 (33.3)
**Case–control**				
Zhang et al. (2020) China	Case control, *n* = 190 Int: Umifenovir 200 mg three times daily for 5–10 days Ctr: Oseltamivir 75 mg once daily or placebo	NR	Number of individuals with positive COVID-19 diagnosis, *n* (%) Int: 2 (2) Ctr: 19 (21) (Odds ratio: 0.011, 95% CI: 0.001–0.125, *p* = 0.003)	NR
Zhou et al. (2020) China	Case–control, *n* = 238 Int: Arbidol	Mild and severe	Median duration of SARS-CoV-2 virus shedding: 23 days (IQR, 17.8–30 days) SARS-CoV-2 RNA clearance was significantly delayed in patients who received Arbidol > 7 days after illness onset, compared with those in whom Arbidol treatment was started ≤ 7 days after illness onset (HR, 1.738 [95% CI, 1.339–2.257], *P* < 0.001).	NR
**Case series**				
Chen H. et al. (2020) China NCT04291729	Case series, *n* = 11 Int: Danoprevir 100 mg twice daily and ritonavir 100 mg twice daily ± with interferon-α2b atomization inhalation (5 million units) twice daily for 4–12 days with	Moderate	Use of danoprevir with ritonavir appears to be safe and effective in supressing the viral replication of SARS-CoV-2. Median days to negative SARS-CoV-2 detection, (range): 2 (1–8) Median days to improvement in chest CT scans, (range): 3 (2–4)	NR
Gautret et al. (2020) France	Case series, *n* = 80 Int: Hydroxychloroquine and azithromycin over a period of at least 3 days	Mild	Mean length of infectious disease unit stay before discharge: 5 days All patients improved clinically except one 86-year-old patient who died, and one 74-year-old patient still in intensive care. Observations: - Rapid fall of nasopharyngeal viral load was noted: 83% negative at Day 7, and 93% at Day 8. - Virus cultures from patient respiratory samples were negative: 97.5% of patients at Day 5.	Nausea or vomiting: 2.5% Diarrhea: 5.0% Blurred vision: 1.2%
Haerter et al. (2020) Germany	Case series of PLWH with COVID-19, *n* = 17 out of 33 with tenofovir use in combination with: - Bictegravir/emtricitabine (*n* = 6) - Rilpivirine/emtricitabine (*n* = 3) - Darunavir/cobicistat/emtricitabine (*n* = 3) - Elvitegravir/cobicistat/emtricitabine (*n* = 3) - Nevirapine/emtricitabine (*n* = 2)	All mild except two critical and one severe	All recovered, one death (critical)	NR
Holshue et al. (2020) United States	Case report, *n* = 1 Remdesivir (dose and frequency not reported)	NR	Improvement reported in patient condition	NR
Lim et al. (2020) South Korea	Case report, *n* = 1 Lopinavir–ritonavir (400 mg and 100 mg) twice daily for 10 or more days	Mild	Reduced viral loads and improved clinical symptoms with treatment of antiviral	NR
Liu et al. (2020) China	Case series, *n* = 10 Int: Lopinavir 400 mg twice daily with interferon-α2b atomization inhalation (5 million unit) twice daily	Mild, moderate and severe	Use of lopinavir appears to be effective	Any adverse event (any grade), *n* (%) Int: 3 (30%)
Wang Z. et al. (2020) China	Case series, *n* = 4 Umifenovir 200 mg three times daily, lopinavir–ritonavir (400 mg and 100 mg) twice daily with traditional Chinese medicine for 6–15 days with supplemental oxygen	Mild, severe	Improvement reported in three patients, of which two were confirmed SARS-CoV-2 negative.	
Young et al. (2020) Singapore	Case series, *n* = 18 Int: Lopinavir–ritonavir (200 mg and 100 mg) twice daily for up to 14 days and supportive therapy ± supplemental oxygen, *n* = 5 Ctr: Supportive therapy ± supplemental oxygen	Mild and moderate	Equivocal improvements between both groups	Adverse effect (all) Int: 4 (80%)
**Other study types**				
Xi et al. (2020) China	Retrospective single-group study (*n* = 94). All patients were treated with Arbidol (100 mg TDS for 14 days) and moxifloxacin (0.4 g once a day for 7–14 days).	27 severe (ICU) and 57 “ordinary warded” patients	After treatment of Arbidol and moxifloxacin for 1 week, the rates of SARS-CoV-2 nucleic acid turning negative were 69.2% in the severe group and 77.8% in the ordinary group.	NR
Zuo et al. (2020) China	Retrospective cross-sectional, *n* = 181 Either lopinavir/ritonavir or lopinavir/ritonavir + IFN-α or lopinavir/ritonavir + IFN-α + Arbidol (dose, frequency not reported)	Mild, moderate and severe	Median duration of viral shedding 18.0 days (IQR 15.0–24.0) Median length of hospital stay: 17.0 days (IQR 14.0–21.0) Median time from illness onset to discharge: 23.0 days (IQR 19.0–28.5)	NR

*Ctr, control; Int, intervention; NR, not reported; PLWH, people living with HIV.*

Most of the registered trials were very small in size with sample size of fewer than 100 patients, with a median sample size of 145 (IQR: 60–343). In most trials, participants had to be aged 18 years and above. Most of these trials were in the recruiting stages (*n* = 39) or the preparation stages (*n* = 19). There was only limited data available on the efficacy of antivirals on COVID-19 and their clinical impact. Most of the trials will examine a myriad of primary outcomes, including time to clinical improvements, number of individuals requiring mechanical ventilation, number of individuals hospitalized into ICU, length of hospitalization, mortality as well as absence of virological indicators. Three studies also used physical functioning scores based upon an ordinal 7-point scale from the WHO master protocol and the National Early Warning Score 2 (NEWS2).

## Direct Antivirals Used in Covid-19

### Protease Inhibitors

Successful entry of the SARS-CoV-2 into the cell will depend on the activation of envelope glycoprotein by host cell protease. As such, protease enzyme inhibitors are considered an excellent drug target for patients with COVID-19 (Table 3). Examples of such drugs include lopinavir, ritonavir, darunavir, danoprevir and the experimental drug ASC-09. Among these agents, the most commonly examined protease inhibitor was lopinavir/ritonavir combination, using a dosing regimen of 400 mg/100 mg lopinavir/ritonavir twice daily for up to 14 days which was reported in 18 published studies (Cai et al., 2020; Cao et al., 2020; Deng et al., 2020; Giacomelli et al., 2020; Kim et al., 2020; Li et al., 2020; Lim et al., 2020; Liu et al., 2020; Shi et al., 2020; Vizcarra et al., 2020; Wang Y. et al., 2020; Yan et al., 2020; Ye et al., 2020; Young et al., 2020; Zhu et al., 2020b; Zuo et al., 2020). These studies were conducted in China (*n* = 13), South Korea (*n* = 2), Italy, Singapore, and Greece (*n* = 1 each). Results from the published randomized controlled studies suggest that there is limited clinical efficacy of the combination (Cao et al., 2020; Hung et al., 2020; Li et al., 2020; Zheng et al., 2020b). In a recently completed RCT in China, the lopinavir/ritonavir combination was reported to have limited efficacy, with no difference in time to clinical improvement (median, 16 days), duration of intensive care unit stay, days of mechanical ventilation, or days of oxygen support (Cao et al., 2020). Authors reported that there appears to be some benefit when patients were given the drug therapy earlier (within 12 days of symptom onset) as they experienced a shorter time to clinical improvement (HR 1.25; 1.77–2.05 versus 1.30; 0.84–1.99). Nevertheless, given the significant drug-drug interaction and potential risk of adverse events including gastrointestinal distress such as nausea and diarrhea and hepatotoxicity, caution should be exercised while using this combination given that nearly 20% to 30% of patients have elevated transaminases at presentation (Wu and McGoogan, 2020; Wu C. et al., 2020).

**TABLE 3:** Overview of mechanism of action of antivirals and recommended doses for use in COVID-19 patients.

Antiviral	Mechanism of action	Recommended dosing regimen	Contraindication	Adverse effects	Drug interactions
**Broad spectrum antiviral**					
Triazavirin	Guanosine nucleotide analog that inhibits RNA synthesis. The drug was developed as a potential treatment for influenza A and B, including the H5N1 strain. Triazavirin also showed activity against tick-borne encephalitis virus, forest-spring encephalitis virus in animal models.	Not available	Not available	Gastrointestinal effect	Not available
Umifenovir	Indole derivative which has dual mechanism- direct acting virucidal activity and inhibiting several stages of viral life cycle, such as virus entry, membrane fusion and viral replication. It is currently licensed in China and Russia for the prophylaxis and treatment of influenza and other respiratory viral infections. It inhibits *in vitro* hepatitis C virus, Ebola virus, Zika virus, West Nile virus, and tick-borne encephalitis virus.	200 mg every 8 h for 7 to 14 days.	Children under 2 years	Allergic reaction, gastrointestinal upset, elevated transaminases	Inducers and inhibitors of CYP3A4
**RNA−dependent RNA polymerase (RdRP) inhibitor**					
Favipiravir	Pyrazinecarboxamide derivative that mimics purines or purine nucleosides and selectively inhibits RNA-dependent RNA polymerase of RNA viruses during viral replication. Favipiravir showed promising *in vitro* antiviral activities against various RNA viruses, including influenza virus, West Nile virus, Ebola virus, yellow fever virus, and Chikungunya virus. It was approved in Japan in 2014 to treat novel or re-emerging pandemic influenza virus infection when other antiviral drugs are ineffective.	A higher end of the dosing range using a loading dose of 2400 mg to 3000 mg every 12 h × 2 doses followed by a maintenance dose of 1200 mg to 1800 mg every 12 h	Pregnancy, breastfeeding	Hyperuricemia, diarrhea, elevated transaminases, decreased neutrophil count, decreased appetite	CYP2C8 and aldehyde oxidase inhibitor Influenza virus vaccine (live/attenuated)
Remdesivir	Adenosine nucleotide analog and inhibitor of RNA-dependent RNA polymerase. Drug was initially developed to treat Ebola and Marburg virus infections. It has demonstrated *in vitro* and *in vivo* activity in animal models against coronaviruses including MERS and SARS.	200 mg loading dose, and 100 mg every 24 h as IV infusion.	Not recommended in patients with GFR < 30	Elevated transaminase, kidney injury, hyperglycemia, fever	Chloroquine, hydroxychloroquine
**Protease inhibitor**					
ASC-09 (TMC-310911)	Protease inhibitor that is structurally similar to its parent molecule, darunavir. It acts as a peptidomimetic inhibitor and dimerization inhibitor, inhibits the cleavage of polypeptides into functional proteins required for infectious HIV. It is given in combination with ritonavir.	Ritonavir/ASC-09 100 mg/300 mg twice daily	Allergic to components of ASC-09/ritonavir tablet	Fatigue, nausea, gastrointestinal effects, increase in liver enzyme level	Not available
Danoprevir	Hepatitis C virus NS3 protease inhibitor which selectively inhibits HCV replication. It is used in combination with ritonavir. Danoprevir is currently licensed in China for the treatment of chronic hepatitis C, in combination with ritonavir, peg-interferon alpha and ribavirin.	Danoprevir/ritonavir 100/100 mg twice daily	Not available	Neutropenia	Strong inhibitor of CYP3A4
Darunavir	Protease inhibitor which inhibits HIV-1 protease. It selectively inhibits the cleavage of polypeptides in infected cells, thus preventing the formation of mature viral particles. It is used in combination with cobicistat or ritonavir, which are potent inhibitors of CYP3A isozymes, to increase the systemic exposure of protease inhibitor.	Darunavir/cobicistat 800 mg/150 mg once daily	Severe (Child-Pugh Class C) hepatic impairment, co-administration with CYP3A4 inhibitors	Skin rash, increased serum cholesterol, increased serum glucose, gastrointestinal effect, headache, fatigue, increased liver enzymes	Strong inhibitor and inducer of CYP3A4
Lopinavir/ritonavir	HIV protease inhibitor which selectively inhibits the cleavage of polypeptides in infected cells, thus preventing the formation of mature viral particles. Ritonavir is mainly used to enhance the action of protease inhibitor by inhibition of CYP3A4 isozymes.	400 mg/100 mg every 12 h for up to 14 days	Hypersensitivity, co-administration with CYP3A4 inducer or inhibitor	Gastrointestinal intolerance, nausea, vomiting, diarrhea, pancreatitis, hepatotoxicity, cardiac conduct abnormalities	Inducers and inhibitors of CYP3A4
**Nucleoside inhibitor**					
Azvudine	Azidocytidine nucleoside analog and nucleoside reverse transcriptase inhibitor. It is metabolized intracellularly into active triphosphate form and incorporates into primer strand by reverse transcriptase, resulting viral DNA chain termination. It demonstrates antiviral activity on HIV, hepatitis B virus and hepatitis C virus.	Azvudine 10 mg on day 1, then 5 mg once daily on day 2–5	Not available	Not available	Not available
Tenofovir disoproxil fumarate	Adenosine nucleotide analog and inhibitor of RNA-dependent DNA polymerase resulting in inhibition of viral replication. It is approved for treatment of Hepatitis B and HIV-1 infection.	Tenofovir disoproxil fumarate/emtricitabine 245 mg/200 mg daily	Hypersensitivity	Pruritus, increased serum lipid, gastrointestinal effect, insomnia, pain, dizziness, depression, decreased bone mineral density	Cidofovir, lopinavir/ritonavir, didanosine, atazanavir
Ribavirin	Guanosine nucleoside analog and inhibitor of virus RNA polymerase activity. It is indicated for treatment of chronic hepatitis C virus infection.	500–600 mg twice daily	Pregnancy, hemoglobinopathies, concomitant use with didanosine, CrCl < 50 mL/min	Fatigue, pyrexia, myalgia, headache, depression, hepatic decompensation	Nucleoside analogs, azathioprine
**Neuroamidase inhibitor**					
Oseltamivir	Potent inhibitor of influenza virus neuraminidase enzymes found on the surface of the virus, which prevents budding from the host cell, viral replication, and infectivity. It is currently licensed for used in the treatment and prophylaxis of infection with influenza viruses A (including pandemic H1N1) and influenza B.	75 mg twice daily	Hypersensitivity to oseltamivir or component of the formulation, not recommended in ESRD not undergoing dialysis	Gastrointestinal effect, headache, pain	Dichlorphenamide, probenecid, influenza virus vaccine (live/attenuated)
**Polymerase acidic endonuclease inhibitors**					
Baloxavir Marboxil	Selective inhibitor of influenza cap-dependent endonuclease thus preventing polymerase function and influenza virus mRNA replication. The drug is currently approved for treatment of influenza virus A and B.	80 mg on day 1, day 4 and day 7 (no more than 3 doses)	Hypersensitivity	Diarrhea, bronchitis, nausea, sinusitis, headache	Polyvalent cation-containing laxatives, antacids or oral supplements Live attenuated influenza virus

While there are no RCTs on the other protease inhibitors darunavir and danoprevir, real world evidence have been reported from Germany and China (Chen H. et al., 2020; Haerter et al., 2020; Shi et al., 2020). Haerter et al. (2020) in Germany reported the outcomes of a case series of patients living with HIV treated with antiretroviral treatment including darunavir. Of the four patients treated, one died while the other three patients recovered. Shi et al. (2020) similarly reported in their case series on the limited efficacy of darunavir in terms of reducing duration from illness onset to admission and clinical symptoms. Only one small study reported the safety of danoprevir in patients with COVID-19. However, taken together these data are difficult to interpret given the concomitant use of drug therapies, lack of comparator treatment and heterogeneity of disease severity.

### Broad Spectrum Antiviral

Another drug commonly examined is umifenovir, a broad spectrum antiviral licensed in China and Russia for influenza. Umifenovir prevents viral host cell entry by inhibiting the membrane fusion of the viral envelope and host cell cytoplasmic membrane (Blaising et al., 2014; Fink et al., 2018; Haviernik et al., 2018). The drug was suggested to have some effects in reducing the risk of COVID-19 transmission and has been examined for post-exposure prophylaxis using a dose of 200 mg orally every 8 h. In an early pilot study from China, treatment with umifenovir was found to reduce SARS-CoV-2 viral loads, with 94% of patients treated with umifenovir reported negative SARS-CoV-2 viral load compared to 53% in the control (Deng et al., 2020). Nevertheless, the results from two RCTs suggested limited efficacy in treating COVID-19 (Chen C. et al., 2020; Li et al., 2020), as the recovery rates were comparable with control.

### RNA−Dependent RNA Polymerase (RdRP) Inhibitor

Favipiravir is another oral antiviral that has been examined recently. Favipiravir is a pyrazinecarboxamide derivative and guanine analog which selectively inhibits the RNA-dependent RNA polymerase (RdRP) of RNA viruses (Furuta et al., 2009). RdRP is required during the replication process of RNA viruses as it determines the replication rates and mutation of the virus to adapt to the new host environment, which ultimately influences its fidelity. As such, targeting of RdRP has become another mainstay in the treatment of SARS-CoV-2. In a pilot pre-post study in China, 80 patients with COVID-19 were treated with favipiravir with a loading dose of 1600 mg followed by a maintenance dose of 600 mg three times daily for up to 14 days. After 14 days of treatment, the authors found that patients treated with favipiravir had better treatment outcomes in terms of disease progression and viral clearance compared to those treated with lopinavir/ritonavir (Cai et al., 2020). Two recently completed RCTs in China had reported promising clinical results due to the higher 7-day recovery rates, and symptom improvements such as fever and cough (Chen C. et al., 2020; Lou et al., 2020). With no significant adverse events were reported, favipiravir is currently being examined in several clinical trials as a potential target drug for SARS-CoV-2.

Remdesivir is another nucleotide analog inhibitor of RdRP that have been extensively examined as a potential anti SARS-CoV-2 medication. The earliest report on the use of remdesivir was reported by Holshue et al. (2020), which reported improvement in the patient’s condition after treatment. Since then, two RCTs on remdesivir has been conducted using a dose of remdesivir 200 mg on day 1, followed by 100 mg daily for up to 10 days. In the first RCT of 237 patients with COVID-19 by Wang Y. et al. (2020) in China, the authors found that more patients on remdesivir had clinical improvements after 28 days, and they reported faster time to symptoms improvements compared to control. Beigel et al. (2020) meanwhile reported a large multi-center RCT in Europe, Asia, and America on 1,107 patients treated with either remdesivir or placebo for 10 days. In their study, they found that the median time to recovery was much faster with remdesivir treatment, with a significantly higher number of patient who recovered. Nevertheless, there are uncertainties about the adverse effects of the drugs, and more clinical trials are underway to examine the potential of this drug in SARS-CoV-2.

### Nucleosidase and Neuroamidase Inhibitors

Another class of drugs that has been used in SARS-CoV-2 is the neuroamidase inhibitors such as oseltamivir. Given that the COVID-19 outbreak in China occurred during the peak influenza season, a large proportion of patients had received oseltamivir therapy prior to the discovery of SARS-CoV-2 as these agents have been used for various influenza subtype and other RNA viruses to inhibit the spread of the influenza virus (Wang et al., 2014; Malosh et al., 2018). Several clinical trials are currently evaluating the effectiveness of oseltamivir either alone or as a combination such as with chloroquine and favipiravir, but given its pharmacological action, there is limited role of these drugs in the management of COVID-19 once influenza has been excluded.

Similarly, the neuroamidase inhibitors ribavirin and azvudine have been recommended in the initial stages for management of COVID-19, given that the symptoms were thought to be due to pneumonia. There is currently no evidence to suggest that ribavirin when used alone offers any benefit in the management of COVID-19. The combination therapy of ribavirin, lopinavir/ritonavir and interferon beta-1b was recently shown to have some positive results and would need to be explored further (Hung et al., 2020). However, as ribavirin causes a dose-dependent hematological toxicity, and is a known teratogen, there is limited value of this drug in the treatment of COVID-19.

### Polymerase Acidic Endonuclease Inhibitors

The only drug in its class examined identified in the current review was baloxavir marboxil. This drug targets the viral polymerase acidic protein to block the endonuclease function, resulting in the inhibition of virus mRNA transcription and infection (Koszalka et al., 2019; Locke et al., 2019). Only one small clinical study in China has been identified in the current review, but due to the small sample the implications will be limited (ChiCTR2000029548).

## Discussion

With no therapeutic agent is currently known to be effective for COVID-19, multiple different antivirals have been examined based upon the early *in vitro* evidence against SARS-CoV. While several case series and reports showed improvements with use of lopinavir-ritonavir, the recently published study by Cao et al. (2020) have showed limited benefits highlighting the difficulty in finding an appropriate agents for rapid implementation in such outbreaks. It remains unfortunate that this therapy is ineffective, given that this would have represented an immediate and safe oral therapy for COVID-19. For most of the current trials reported, these are underpowered and unlikely to provide the healthcare community with the necessary high quality evidences needed to combat this pandemic if taken individually. In addition, most of the trials registered will only include patients aged 18 and above, and thus will unlike to provide the necessary information on children, adolescents, pregnant women or even those with respiratory diseases (Lee, 2020).

These trials also included a wide range of primary outcomes including time to clinical improvements, number of individuals requiring mechanical ventilation, number of individuals hospitalized into ICU, length of hospitalization, mortality as well as absence of virological indicators. As most of the outcomes that will be reported varied, and will include subjective outcomes, this may lead to measurement bias. Importantly, few of the current trials have reported on mortality in their study either as a primary or secondary outcome. While the case fatality rates differs between countries, ranging from as low as 0.3% to as high as 11.0%, these reports have not been forthcoming in all the included studies and should be given attention (Rajgor et al., 2020). In addition, most of the current studies are not coordinated, leading to inconsistencies among trials in their definitions of conditions and inclusion criteria, the design and delivery of intervention and comparison, as well as measurement of the outcomes. Cognisant of this, the World Health Organization (WHO) is initiating a clinical trials experts group which will aim to develop a master protocol for a RCT to evaluate efficacy of therapeutics against nCov (World Health Organization, 2020). Other impending initiatives include the strengthening of management and coordination of the promising drugs such as remdesivir and favipiravir, which should be prioritized for clinical studies. This is based upon the potential activity of both agents against RNA polymerase, established use in novel influence and also oral bioavailability. This ideally should involve the pharmacist who can help in the development of treatment protocols, monitoring of drug adverse events as well as assist in the expanded access of these new investigational drugs (Lee et al., 2019; Stevens et al., 2020).

Investigators should also consider using other clinical trial designs including step-wedge design which may reduce the need for large sample sizes (Baio et al., 2015). In addition, a database should also be setup to share all available existing data between sites and countries, which effectively create a real-world evidence study network, which can increase the speed of information dissemination especially in pandemics such as COVID-19. Indeed, there is a need for researchers to report as much details as possible to ensure reproducibility of results especially as these studies currently use very weak outcomes which can limit the efficacy assessment. Nevertheless, the development of clinical trials during an outbreak is an adaptive process, with new evidence being generated at an impressive rate. As such, we believe that these results generated will inform the adaptation of existing and new trials that are being developed. Indeed, with progressive release of trial results, there is a need for a living systematic review to progressively update the pooled results with each additional trial included. This is crucial in view of the small sample size of individual studies.

Nevertheless, we acknowledge that there are some limitations to this review, given that we had only one reviewer who had conducted the search. In addition, this review also included several pre-print articles which have not been peer-reviewed and thus may not provide the academic rigor normally required for published studies. However, in view of the evolving situation of COVID-19 and the need for rapid understanding of this disease, the decision to include these studies were needed in order for us to provide the readers with the most updated information available. Most of the published treatment data to date are derived from observational studies which have relatively small sample size, which may introduce risk regarding the magnitude of effect sizes.

In summary, this updated review of antivirals in COVID-19 showed that there is limited information available to guide clinical practice as well as the need for a more coordinated research network to seek the best therapeutic options especially in pandemics. While several agents reviewed have suggested some potential benefits of therapy, the evidence remains inconclusive.

## Author Contributions

SL conceived the study, conducted the analysis, and wrote the draft. ST, YL, and NL collected the data, conducted the analysis, and edited the draft. All authors approved the final draft.

## Conflict of Interest

The authors declare that the research was conducted in the absence of any commercial or financial relationships that could be construed as a potential conflict of interest.
