# Supplementary material for: Directly Acting Antivirals for COVID-19: Where Do We Stand?
Source: Front Microbiol. 2020 Aug 5;11:1857. doi: 10.3389/fmicb.2020.01857 (PMC7419656; doi:10.3389/fmicb.2020.01857)

Table 1: Study Characteristics of studies that are registered onto the clinical trial registry databases

Date of Registration	Trial ID	Recruiting Status	Study design, sing/multi-centre, targeted participant number	Country	Intervention Group(s)	Comparison Group(s)	Primary Outcomes	Importance of study
**Protease inhibitors**								
23/1/2020	ChiCTR 2000029308	Recruiting	RCT (2-arm), single centre, N=160	China	Lopinavir-ritonavir (400 mg /100 mg) twice a day	Conventional standardized treatment	• Clinical improvement time<br>• Time to clinical improvement<br>• Mortality on day 28<br>• Duration of mechanical ventilation/ oxygen therapy/ hospitalization (days)	Medium
29/1/2020	ChiCTR 2000029387	Recruiting	RCT (3-arm), single centre, N=78	China	Lopinavir/ ritonavir, ribavirin and interferon alpha 1b<br>Ribavirin with interferon alpha 1-b	Lopinavir/ ritonavir with interferon alpha 1b	• Time to negative viral load	Low
2/2/2020	ChiCTR 2000029468	Not yet recruiting	Case-control, Single, N=120, 2 arm	China	Lopinavir/ritonavir with emtritabine/tenofovir disoproxil fumarate tablets	Lopinavir/ritonavir	• Survival rate	Low
3/2/2020	ChiCTR 2000029539	Recruiting	RCT (2-arm), single centre, N=328	China	Lopinavir/ritonavir	Usual care	• The incidence of adverse outcome within 14 days after admission:<br>• Respiratory rate ≥ 24 times / min in the state of resting without oxygen inhalation	Medium
5/2/2020	NCT04252885	Recruiting	RCT (3-arm), single centre, N=125	China	Lopinavir/Ritonavir 400/100mg twice daily for 7-14 days<br>Umifenovir 200mg three times daily for 7-14 days	Standard care	• The rate of virus inhibition<br>• The disease prorogation- temperature<br>• The disease prorogation- respiratory function<br>• The disease prorogation- respiratory function<br>• - The disease prorogation- respiratory function	Low

6/2/2020	ChiCTR2000029600	Recruiting	Non-randomized, single centre, N=90	China	Lopinavir/ritonavir with alpha-Interferon atomization<br>Favipiravir with alpha-Interferon atomization	Alpha-Interferon atomization	• Time to negative nCoV<br>• Adverse events	Low
5/2/2020	NCT04255017	Recruiting	RCT (4-arm), single centre, N=400	China	Lopinavir/ ritonavir 400/100mg twice a day for 14 days<br>Oseltamivir 75mg twice a day for 14 days<br>Umifenovir 200mg three a day for 14 days	Symptomatic supportive treatment	• Duration of clinical improvement, defined as no fever, cough or other symptoms with improved lung CT, SPO2> 93% or PaO2/FiO2>300mmHg<br>• Time to lung imaging recovery	High
5/2/2020	NCT04251871	Recruiting	RCT (2-arm), single centre, N=150	China	Oxygen therapy, alfa interferon via aerosol inhalation, and lopinavir/ ritonavir with TCM granules	Oxygen therapy, alfa interferon via aerosol inhalation, and lopinavir/ ritonavir	• Time to complete remission of 2019-nCoV infection-associated symptoms<br>• The incidence of dyspnea with low oxygen saturation level and high respiratory rate<br>• Number of subjects who develop complications of 2019-nCoV infection<br>• Time to improvement of abnormalities in chest radiology	Low
11/2/2020	ChiCTR2000029741	Recruiting	RCT (2-arm), single centre, N=112	China	Lopinavir / Ritonavir	Chloroquine Phosphate	• Hospital length of stay<br>• All cause mortality at 28 days<br>• Oxygenation index during treatment<br>• Viral nucleic acid load	Medium
12/2/2020	ChiCTR2000029759	Suspended	Prospective, Multiple centres, n=60	China	Lopinavir / Ritonavir and IFN aerosol inhalation<br>Umefenovir and IFN aerosol inhalation.	ASC09/ritonavir (ASC09F) and IFN aerosol inhalation	• Time to recovery<br>• Rate of composite adverse outcomes<br>• Rate of undetectable new coronavirus pathogen nucleic acid<br>• Rate of mechanical ventilation	Low
19/2/2020	NCT04275388	Not yet recruiting	RCT (2-arm), multicentre, N=348	China	Lopinavir / ritonavir, alpha-interferon nebulization	Xiyanping injection	• Clinical recovery time<br>• Time to negative virus detection<br>• Incidence of severe or critical pneumonia	Medium

19/2/2020	ChiCTR2000030000	Recruiting	RCT, single centre, N=50	China	Ritonavir<br>Novaferon injection<br>TCM + spray inhalation	Interferon alpha	• Rate of composite adverse outcomes: SpO2, PaO2/FiO2, respiratory rate	Low
24/2/2020	ChiCTR2000030187	Completed	RCT (2-arm), single centre, N=60	China	Lopinavir/Ritonavir	Routine symptomatic supportive treatment	• Endotracheal intubation rate at 14 days<br>• Mortality rate within 30 days	Low
24/2/2020	ChiCTR2000030166	Not yet recruiting	RCT (2-arm), single centre, N=20	China	Lopinavir/ritonavir with recombinant human interferon alpha 2b injection) + Qing-Wen Bai-Du-Yin formula granules	Routine treatment	• Nucleic acid detection of throat secretion<br>• Adverse events	Low
25/2/2020	ChiCTR2000030021, NCT04295551	Not yet recruiting	RCT (3-arm), single centre, N=80	China	Lopinavir/ritonavir tablets with Xiyanping injection	Ritonavir treatment	• Clinical recovery time<br>• The time from study drug use to complete fever reduction and cough recovery is measured in hours.<br>• Pneumonia severity index (PSI) score	Low
25/2/2020	ChiCTR2000030218	Recruiting	Retrospective observational, single centre N=80	China	Lopinavir / ritonavir tablets combined with Xiyanping injection	Lopinavir / ritonavir tablets	• Clinical recovery time<br>• Pneumonia Severity Index (PSI) score	Low
27/2/2020	NCT04286503	Not yet recruiting	RCT (4-arm), multicentre, N=520	China	Lopinavir/ ritonavir 400mg/100mg twice daily<br>Umifenovir 200mg three time daily<br>Chloroquine phosphate 500mg twice daily for 7-10 days	Carrimycin 400mg daily for 7-14 days	• Fever to normal time (day)<br>• Pulmonary inflammation resolution time<br>• Negative conversion (%) of 2019-nCOVRNA in gargle (throat swabs) at the end of treatment	High
8/3/2020	ChiCTR2000030593	Not yet recruiting	Observational, multicentre, N= 150	China	Antiviral (Lopinavir/ritonavir, umifenovir, darunavir / corbistadine and favipiravir)		• Alanine transaminase, total bilirubin levels	Low

13/3/2020	NCT04 307693	Terminated	RCT (2-arm), single centre, N=150	Korea	Lopinavir/ritonavir	Hydroxychloroquine sulfate	• Viral load<br>• Time to clinical improvement<br>• Percentage of patients requiring supplemental oxygen<br>• Time to NEWS2 score of 3 or more maintained for 24 hours by day 7<br>• Mortality<br>• Adverse effects	Medium
23/3/2020	ChiCTR 2000031196	Recruiting	Observational, single-centre, N=90	China	Lopinavir/ritonavir and interferon	Routine medical treatment	• Time till the SARS-CoV-2 clearance<br>• Novel coronavirus nucleic acid clearance rate<br>• Adverse reaction rate	Low
25/3/2020	NCT04 321174	Not yet recruiting	RCT (4-arm), multicentre, N=1220	Canada	Lopinavir/ ritonavir 400/100mg twice a day for 14 days	Usual care	• Microbiologic evidence of infection<br>• Adverse events<br>• Days of hospitalization attributable to COVID-19 disease<br>• Respiratory failure requiring ventilator support<br>• Mortality<br>• Short-term psychological impact of exposure to COVID-19 disease<br>• Long-term psychological impact of exposure to COVID-19 disease<br>• Health-related quality of life	High
26/3/2020	NCT04 321993	Recruiting	RCT (5-arm), multicentre, N=1000	Canada	Lopinavir/ ritonavir 400/100mg twice a day for 10 days<br>Hydroxychloroquine 400mg twice a day for 10 days<br>Baricitinib 2 mg daily for 10 days<br>Sarilumab 200mg subcutaneous injection once	Standard of care	• Clinical status of subject using a 6 point scoring system<br>• Death	High

1/4/2020	NCT04 330690	Recruiting	RCT (4 arm), multicentre, N=2900	Canada	Lopinavir/ritonavir (400 mg/100 mg) for 14-days plus supportive care<br>Hydroxychloroquine 800mg twice daily for 1 day then 400mg twice daily for 10 days plus supportive care<br>Remdesivir 200mg on day 1, 100 mg daily for 9 days plus supportive care	Standard supportive care	• All cause mortality	High
15/4/2020	NCT04 346147	Recruiting	RCT, multicentre, n=165	Spain	Hydroxychloroquine 200mg twice daily + imatinib 400mg daily<br>Hydroxychloroquine 200mg twice daily + baricitinib 4mg daily	Hydroxychloroquine 200mg twice daily + lopinavir / ritonavir (200/50 mg) twice daily	• Time to clinical improvement	Low
27/5/2020	NCT04 403100	Recruiting	RCT (4 arm), multi-centre, n=1968	Brazil	Hydroxychloroquine 400mg<br>Litonavir/ritonavir (400/100mg) twice daily<br>Litonavir/ritonavir (400/100mg) twice daily with hydroxychloroquine 400mg	Placebo	• No. of participants hospitalized for progression of COVID-19 disease<br>• -Death due to COVID-19	Medium
26/2/2020	ChiCTR 200003 0259	Recruiting	RCT (2-arm), single centre, N=60	China	Danoprevir/ritonavir 100/100 mg twice daily	Standard of care	• Rate of composite adverse outcomes: SpO2, PaO2/FiO2, respiratory rate	Low
3/3/2020	ChiCTR 200003 0472	Recruiting	Prospective Cohort, single centre, n=20	China	Danoprevir/ ritonavir with conventional treatment	Conventional treatment	• Rate of composite adverse outcomes: SPO2, PaO2/FiO2 and respiratory rate<br>• Clinical remission rate and time<br>• Novel coronavirus nucleic acid detection	Low
3/2/2020	ChiCTR 200002 9541	Not yet recruiting	RCT (3-arm), single centre, N=100	China	Darunavir/Cobicistat (800mg/150mg) daily with thymosin 1.6mg daily<br>Lopinavir/ritonavir	Thymosin 1.6mg daily	• Viral load	Medium

					(400mg/100mg) twice daily with thymosin 1.6mg daily			
5/2/2020	NCT04 252274	Recruiting	RCT, single centre, n=30	China	Darunavir, cobicistat daily for 5 days and conventional treatments	Conventional treatment	• Virological clearance rate of throat swabs, sputum, or lower respiratory tract secretions at day 7	Low
6/2/2020	NCT04 261907	Not yet recruiting	RCT (2-arm), single centre, N=160	China	ASC09/ ritonavir group	Lopinavir/ritona vir group	• The incidence of composite adverse outcome<br>• Time to recovery<br>• Rate of mechanical ventilation	Medium
6/2/2020	ChiCTR 200002 9603	Recruiting	RCT (2-arm), multiple centres, N=160	China	Conventional standardized treatment and ASC09/Ritonavir	Conventional standardized treatment and Lopinavir/Riton avir	• The incidence of composite adverse outcome within 14 days after admission<br>• Rate and time of laboratory indicators related to disease improvement	Medium
**RNA - dependent RNA polymerase (RdRP) inhibitor**								
5/2/2020	NCT04 252664	Suspended	RCT (2-arm), single centre, N=308	China	Remdesivir 200 mg loading dose on day 1 is given, followed by 100 mg iv once-daily maintenance doses for 9 days	Placebo	• Time to clinical recovery, defined as initiation of study treatment (active or placebo) until normalisation of fever, respiratory rate, and oxygen saturation, and alleviation of cough, sustained for at least 72 hours.<br>• Normalisation and alleviation criteria:	High
6/2/2020	NCT04 257656	Terminate d	RCT (2-arm), single centre, N=453	China	Remdesivir 200 mg loading dose on day 1 is given, followed by 100 mg iv once-daily maintenance doses for 9 days	Placebo	• A decline of two categories from status at randomisation on a six-category ordinal scale of clinical status which ranges from 1 (discharged) to 6 (death).	High
3/3/2020	NCT04 292730	Active, not recruiting	RCT (4-arm), multicentre, N=1600	USA	Remdesivir 200mg on day 1 and 100mg for 4 days Remdesivir 200mg on day 1 and 100mg for 9 days	Remdesivir 200mg on day 1 and 100mg for 9 days with	• Odds of improvement on a 7-point Ordinal Scale at day 14	High

						mechanical ventilation		
6/3/2020	NCT04 292899	Active, not recruiting	RCT (4-arm), multicentre, N=6000	USA	Remdesivir 200mg on day 1 and 100mg for 4 days<br>Remdesivir 200mg on day 1 and 100mg for 9 days	Remdesivir 200mg on day 1 and 100mg for 9 days with mechanical ventilation	• Odds of improvement on a 7-point Ordinal Scale at day 14	High
10/3/2020	NCT04 302766	Recruiting	Retrospective	USA	Remdesivir (dose, frequency not mentioned)	NR	• NR	Low
20/3/2020	NCT04 315948	Recruiting	RCT (5-arm), multicentre, N=3100	France	Remdesivir<br>Lopinavir/ritonavir<br>Interferon Beta-1A<br>Hydroxychloroquine	Standard of care	• Percentage of subjects reporting each severity rating on a 7-point ordinal scale<br>• The time to discharge or to a NEWS of # 2 and maintained for 24 hours, whichever occurs first.<br>• Number of oxygenation free days in the first 28 days<br>• Hospitalization<br>• Mortality	High
28/4/2020	NCT04 365725	Recruiting	Retrospective study, single centre, N=200	France	Remdesivir		• Clinical course on Day 15	Low
16/3/2020	ChiCTR 200003 0894	Recruiting	RCT (2-arm), multicentre, N=150	China	Favipiravir and tocilizumab<br>Tocilizumab only	Favipiravir only	• Clinical cure rate<br>• Viral nucleic acid test negative conversion rate<br>• Mortality<br>• Hospitalisation days	Low
23/2/2020	ChiCTR 200003 0113	Recruiting	RCT (2-arm), single centre, N=30	China	Favipiravir (dose and frequency not stated)	Litonavir/ritona vir treatment (dose and frequency not stated)	• Blood routine tests results including liver function, renal function, blood gas analysis and chest CT examination	Low
26/2/2020	ChiCTR 200003 0254	Completed	RCT (2-arm, open-label), multicentre, N=240	China	Favipiravir tablets (dose and frequency not stated)	Umifenovir tablets (dose and frequency not stated)	• Clinical recovery rate of day 7	Medium

14/4/2020	NCT04345419	Recruiting	RCT (6 arm), single centre, N=120	Egypt	Favipiravir (dose and frequency not stated)<br>Chloroquine (dose and frequency not stated)<br>Nitazoxanide<br>Ivermection<br>Niclosamide	Any other drugs (oseltamivir/combination of any of above treatment)	• Number of patients with decreased viral load	Low
6/5/2020	NCT04376814	Completed	Quasi-RCT, single centre, n=40	Iran	Favipiravir 1600 mg loading dose and 600 mg three times daily plus 200 mg hydroxychloroquine twice daily for 7 days	Litonavir/ritonavir (200/50mg) twice daily plus 200 mg hydroxychloroquine twice daily for 7 days	• Mortality rate<br>• Long term hospitalisation<br>• Laboratory treatment response<br>• Need for oxygen therapy	Low
**Nucleoside inhibitor**								
5/2/2020	ChiCTR2000029592	Not yet recruiting	Observational, Single-centre, N=1000	China	Umifenovir (dose and frequency not stated)	Usual care	• 2019-nCoV RNA negative	Medium
7/2/2020	ChiCTR2000029621<br>NCT04260594	Recruiting	RCT (2-arm), multicentre, N=380	China	Umifenovir + “basic treatment”	“Basic treatment”	• Virus negative conversion rate in the first week<br>• Virus negative conversion rate<br>• Disease progression rate<br>• Mortality rate<br>• Incidence of severe adverse reactions	Medium
17/3/2020	ChiCTR2000030922	Recruiting	Non randomised, Multicentre, n=30 (target)	China	Umifenovir + ribavirin (dosage not stated)	Ribavarin with long-acting interferon alpha-2a (135ug)	• COVID-19 nucleic acid negative conversion rate<br>• All-cause mortality<br>• Adverse event	Low
17/4/2020	NCT04350684	By invitation only	RCT, single centre, n=40	Iran	Umifenovir + Interferon-β 1a + Lopinavir / Ritonavir + Single Dose of Hydroxychloroquine + Standards care	Interferon-β 1a + Lopinavir / Ritonavir + Single Dose of Hydroxychloroq	• Time to clinical improvement	Low

						uine + Standards care		
11/3/2020	NCT04 303299	Not yet recruiting	RCT (6-arm), single centre, n=320	Thailand	Oseltamivir 300mg with hydroxychloroquine 800 mg daily<br>Darunavir 400 mg every 8 hours, ritonavir 200 mg per day, oseltamivir 300mg per day, with hydroxychloroquine 400mg per day<br>Lopinavir/Ritonavir (800/200) per day with oseltamivir 300 mg per day<br>Lopinavir/Ritonavir (800/200) per day with favipiravir 2400 mg, 2400 mg, and 1200 mg every 8 h on day 1, and a maintenance dose of 1200 mg twice a day<br>Darunavir 400 mg every 8 hours ritonavir 200 mg per day, favipiravir 2400 mg, 2400 mg, and 1200 mg every 8 h on day 1, and a maintenance dose of 1200 mg twice a day plus hydroxychloroquine 400 mg per day	Quarantine treatment	• SARS-CoV-2 eradication time	Low
8/4/2020	NCT04 338698	Not yet recruiting	RCT (8-arm), multi-centre, N=500	Pakistan	Oseltamivir<br>Hydroxychloroquine<br>Azithromycin<br>Hydroxychloroquine with azithromycin	Usual care	• Time to negative COVID-19 detection using RT-PCR with viral load of <150 i.u	Medium

					Hydroxychloroquine with oseltamivir<br>Oseltamivir with azithromycin<br>Hydroxychlroquine, oseltamivir & azithromycin			
6/4/2020	NCT04 334928	Recruiting	RCT (4-arm), multicentre, N=4000	Spain	Tenofovir disoproxil fumarate/emtricitabine (245mg/200 mg) daily<br>Hydroxychloroquine 200 mg once a day<br>Tenofovir disoproxil fumarate/emtricitabine (245mg/200 mg) with hydroxychloroquine 200 mg daily	Placebo	• Number of confirmed symptomatic infections of SARS-CoV-2 (COVID-19)	High
15/2/2020	ChiCTR 200002 9853	Recruiting	RCT (2-arm, open label), single centre, N=20	China	Azvudine 5mg daily	Usual care as per guidelines.	• Time and rate of improvement of respiratory symptoms and signs<br>• Time and rate of pulmonary imaging improvement<br>• Time and rate of change to negative COVID-19 nucleic acid test<br>• Length of hospitalization<br>• Mortality	Low
1/3/2020	ChiCTR 200003 0424	Not yet recruiting	Non-randomised (single-arm), single centre, N=30	China	Azvudine Tablet: D1: 10 mg/day, QD; Starting from D2: 5 mg/day, QD.<br>+<br>Symptomatic treatment		• Sputum/nasal swab/pharyngeal swab/lower respiratory tract secretions were used to detect the negative conversion rate of the new coronavirus nucleic acid (tested daily after two days starting the azvudine tablets) and the negative conversion time	Low

4/3/2020	ChiCTR 2000030487	Recruiting	Non-randomised (single-arm), single centre, N=10	China	Azvudine 10 mg/day at day 1 and 5 mg/day for the next 4 days with symptomatic treatment	Symptomatic treatment	• Negative conversion rate of the new coronavirus nucleic acid<br>• Time to clinical recovery<br>• Pulmonary CT/DR improvement rate<br>• The frequency of respiratory aggravation	Low
Broad spectrum antiviral							•	
19/2/2020	ChiCTR 2000030001	Recruiting	RCT (2-arm), multicentre, N=240	China	Triazavirin	Usual care	• Time to Clinical recovery	Medium
**Polymerase acidic endonuclease inhibitors**								
3/2/2020	ChiCTR 2000029544	Recruiting	RCT (3-arm), single centre, N=30	China	Baloxavir Marboxil tablets with current treatment<br>Favipiravir with current antiviral treatment	Current antiviral treatment	• Time to viral negativity by RT-PCR<br>• Time to clinical improvement<br>• Incidence of mechanical ventilation by day14<br>• Incidence of ICU admission by day14<br>• All-cause mortality by day14, day28<br>• Subjects with viral positive by RT-PCR<br>• Adverse event	Medium
4/2/2020	ChiCTR 2000029548	Not yet recruiting	RCT (3-arm), single centre, N=30	China	Baloxavir Marboxil, 80mg on day1, 80mg on day 4;and 80mg on day7 as necessary with no more than 3 doses of administration in total<br>Favipiravir 600mg tid with 1600mg loading dose for no more than 14 days	Lopinavir-ritonavir (400mg/100mg) twice daily for 14 days	• Viral load<br>• Hospital length of stay or NEWS2<2 for 24 hours	Low
Other antivirals							•	
21/2/2020	ChiCTR 2000030033	Not yet recruiting	RCT (2-arm), single centre, N=828	China	Oral antiviral oral solution (Xiangxue Pharmaceutical)<br>Oral "Wu-Zhi-Fang-Guan-Fang" decoction	Routine quarantine measures	• Proportion of COVID-19 close contacts who have developed as confirmed cases	Medium

1/3/2020	ChiCTR 2000030391	Not yet recruiting	Observational, 2-centre, N=100	China	Antiviral	NA	• Time of virus negative<br>• Improvement rate and time of lung CT at discharge<br>• ICU hospitalisation days	Low
10/3/2020	ChiCTR 2000030703	Recruiting	RCT (2-arm), single centre, N= 40	China	Ixekizumab and antiviral therapy	Antiviral therapy	• Lung function and radiological findings<br>• Blood gas results	Low
18/3/2020	ChiCTR 2000030939	Recruiting	Observational, 2-centre, N=10	China	Oral LL-37 antiviral peptide (CAS001)		• Negative virus detection<br>• Blood results<br>• Adverse events	Low

Figure 1: Number of new trials using antivirals added into trial databases each week (blue bar chart), with the cumulative number (orange)

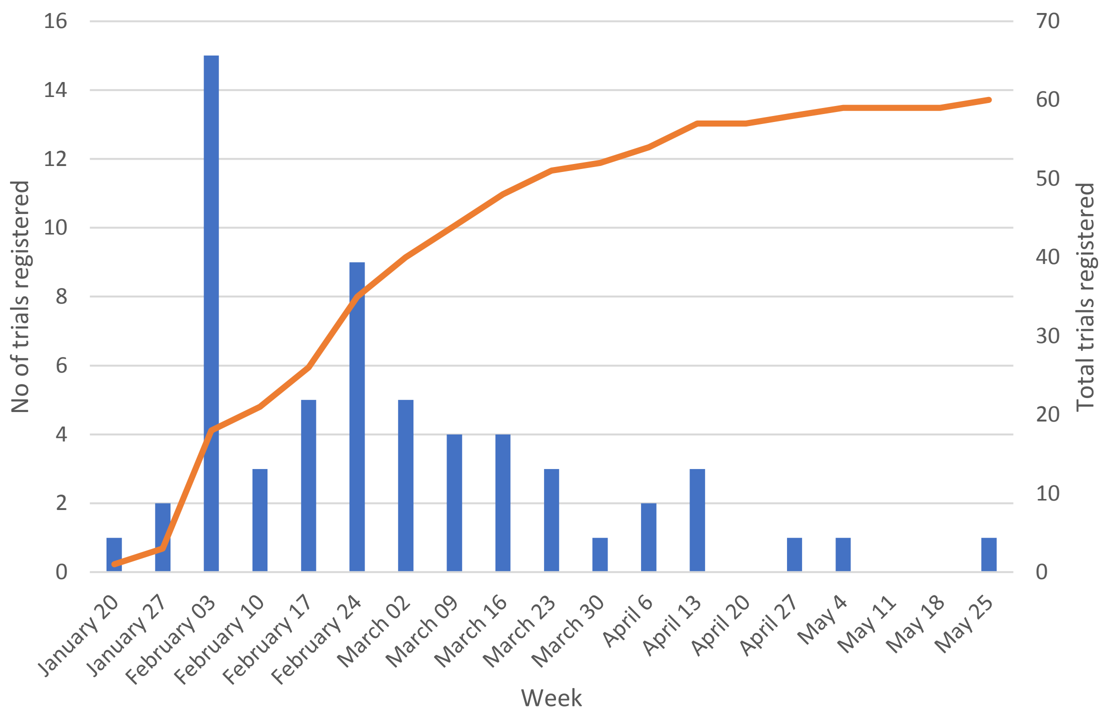

Figure 2: Bubble plot of number of studies registered and total patient. The size of each bubble corresponds to the total number of patients that will be recruited.

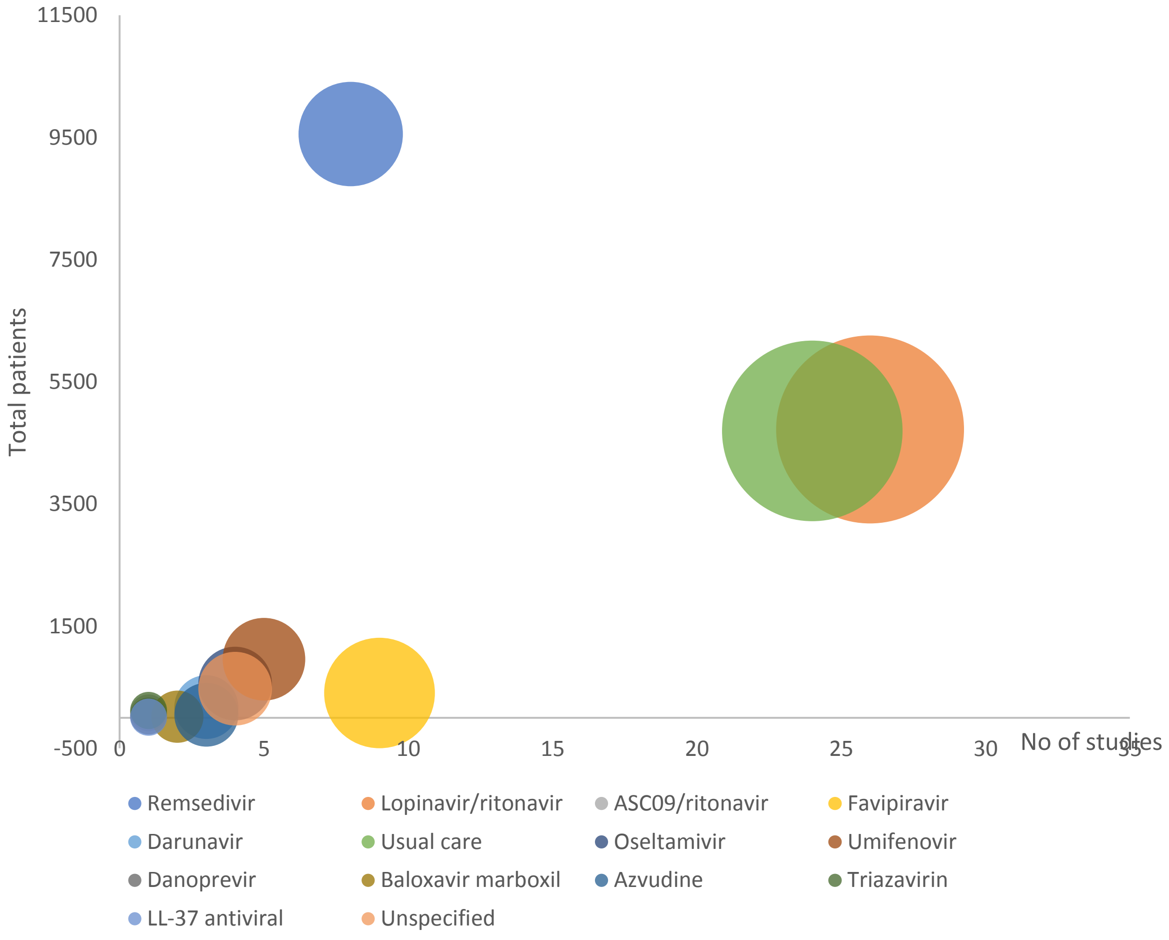

Supplement: Supplementary file 1 [file Data_Sheet_1.pdf]
